# Cross-population metabolome-wide Mendelian randomization study of prostate cancer risk

**DOI:** 10.21203/rs.3.rs-9708129/v1

**Published:** 2026-05-31

**Authors:** Harriett Fuller, Rebecca Rohde, Heather M Highland, Jeffrey Haessler, Lang Wu, Mariaelisa Graff, Elizabeth A Platz, Taryn Alkis, Bing Yu, Eric Boerwinkle, Megan Grove, Charles Kooperberg, Ulrike Peters, Kari E North, David V Conti, Christopher A Haiman, Kristin L Young, Rebecca E Graff, Burcu F Darst

**Affiliations:** Fred Hutchinson Cancer Center; University of North Carolina at Chapel Hill; The University of Texas Health Science Center at Houston School of Public Health; Fred Hutchinson Cancer Center; Louisiana State University Health Sciences Center; University of North Carolina at Chapel Hill; John Hopkins; The University of Texas Health Science Center at Houston School of Public Health; The University of Texas Health Science Center at Houston School of Public Health; The University of Texas Health Science Center at Houston School of Public Health; The University of Texas Health Science Center at Houston School of Public Health; Fred Hutchinson Cancer Center; Fred Hutchinson Cancer Center; The University of Texas Health Science Center at Houston School of Public Health; CU Anschutz; University of California, San Francisco; The University of Texas Health Science Center at Houston School of Public Health; University of California, San Francisco; Fred Hutchinson Cancer Center

## Abstract

**Background:**

Prostate cancer (PCa) is the most common cancer in US men, with Black men experiencing the highest incidence rates and Black and Hispanic men experiencing higher aggressive PCa rates than White men. Metabolomic dysregulation is a cancer hallmark; however, PCa metabolomic epidemiological evidence is heterogeneous and limited in diverse populations.

**Methods:**

We conducted a metabolome-wide two-sample Mendelian randomization (MR) of PCa risk in African (AFR) and European (EUR) genetic ancestry and Hispanic (HIS) ethnicity populations. MR was performed in each population using serum metabolomic genome-wide association study (GWAS) summary statistics from ARIC (N_Metabolites_=250, N_AFR_=1,740, N_EUR_=1,498) and HCHS/SOL (N_Metabolites_=711, N_HIS_=3,166) and PCa GWAS summary statistics from PRACTICAL (N_AFR_=19,391/61,608, N_EUR_=122,188/604,640, N_HIS_=3,931/26,405 cases/controls). MR results were meta-analyzed across populations.

**Results:**

We identified 61 significant associations representing 50 unique metabolites in population-specific or cross-population analyses, with enrichment for lipids, including polyunsaturated fatty acids. Upon evaluating the strength of evidence, considering sensitivity analyses and the consistency of findings across populations, fourteen metabolites had strong evidence, including three drug-modifiable and six dietary-modifiable metabolites. Five were novel metabolites not previously reported in PCa MR studies, including two identified in AFR-specific associations: lysophospholipid 1-linoleoyl-GPE (18:20) and fatty acid hexadecanedioate, with colocalization suggesting a shared causal variant (rs28864441) between hexadecanedioate and PCa, and three identified in cross-population associations: amino acid 3-methoxytryosine, fatty acid 2-hydroxysterate, and peptide gamma-glutamylleucine.

**Conclusions:**

Our findings provide evidence of associations between serum metabolites, particularly fatty acids, and PCa development across populations. The biological mechanisms and clinical utility of these metabolites as biomarkers of PCa risk warrant further investigation.

## Introduction

Among men in the United States, prostate cancer (PCa) is the most commonly diagnosed cancer—accounting for 30% of all new cancer diagnoses—and the second leading cause of cancer-related death. (1) PCa is also one of the most heritable cancers, with an estimated heritability of ~ 58% and well-established genetic factors contributing to PCa risk.(2, 3) The few other established PCa risk factors include older age, a family history of PCa, and race.(4) PCa exhibits the highest rate of health disparities amongst all cancers, with Black and African American men experiencing 1.7 times higher incidence and 2 times higher mortality rates than White men.(4, 5) Furthermore, although Hispanic men experience lower PCa incidence rates than non-Hispanic White men, Hispanic men are more likely to have high-risk PCa and are less likely to undergo PCa screening compared to non-Hispanic White men.(6–9) The underlining factors driving these disparities are not fully understood, but are likely a result of a complex interplay of genetic, environmental and socioeconomic factors.(5, 9, 10)

While midlife prostate-specific antigen has been associated with future risk of developing overall and lethal PCa,(11–14) identifying additional biomarkers could improve our ability to identify individuals with increased PCa risk, potentially informing effective prevention and therapeutic strategies. One rapidly advancing area of research facilitating clinical biomarker identification is metabolomic epidemiology—the study of relationships between metabolites and health-related traits in population-based epidemiologic studies.(15–17) Dysregulation of cellular metabolism is a key cancer hallmark, and multiple prospective investigations have identified metabolites associated with PCa risk.(18–21) For instance, lethal PCa risk has been consistently inversely associated with pre-diagnostic phospholipids important for cell membrane structure and positively associated with pre-diagnostic pyrimidine nucleotides and androgen steroid hormones, which promote normal and cancerous prostate cell growth. (19) However, many PCa metabolite epidemiological findings have not been consistently replicated, which could be partially due to heterogeneity in confounding factors, metabolite levels, metabolite platforms, and statistical methodology across studies. Further, few findings have been consistently reported for risk of overall as opposed to advanced or lethal PCa.(19) This suggests the potential for reverse causation, whereby metabolite associations could be a consequence of the development of PCa rather than metabolites impacting future risk of PCa and disease etiology.(20) An improved understanding of metabolites associated with overall PCa risk may highlight the biological mechanisms underpinning tumorigenesis, furthering our understanding of cancer biology and informing therapeutic development.

Causal inference approaches, such as Mendelian randomization (MR), can address bias due to reverse causation and residual confounding, while uncovering novel etiologic factors, provided specific assumptions are met. MR is particularly useful in metabolomic investigations, where reverse causation could be substantial. In addition, many metabolites are highly heritable (typically ranging from ~ 20–50%), enabling the development of genetic instruments utilized in MR investigations.(22) Several PCa metabolite MR studies have been performed to date and have suggested risk-decreasing associations with phospholipids and risk-increasing associations with nucleotides involved in pyrimidine and purine metabolism,(23–34) complementing observational findings.(21, 35) However, previous PCa metabolomic MR studies were often restricted to small metabolite panels and performed in European descent individuals, limiting the potential for discovery and determining whether findings are generalizable. Previous cross-population metabolite MR studies of other health outcomes have reported that some MR effects differ across populations.(36–38)

To address these gaps in knowledge and investigate the metabolomic underpinnings of PCa risk, we conducted a comprehensive exploratory cross-population two-sample MR study across African (AFR) and European (EUR) genetic ancestry and Hispanic (HIS) ethnicity populations using serum metabolomic genome-wide association study (GWAS) summary statistics, based on a high throughput metabolomics panel, and the largest cross-population PCa GWAS to date. We also performed extensive sensitivity analyses to evaluate MR assumption violations and further investigated metabolites with strong evidence of associations with PCa risk, leveraging colocalization, multivariate MR analyses, and public drug and dietary databases to better understand the potential clinical and public health implications of our findings and guide future research.

## Methods

### Study design and populations

**Figure 1:**
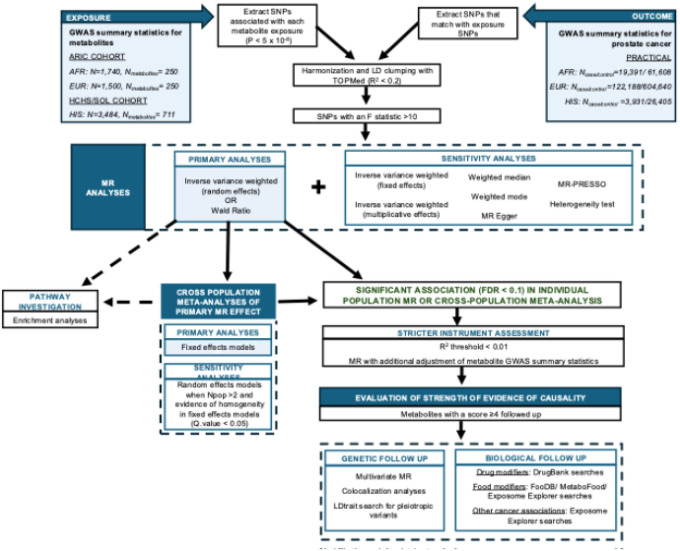
Overview of study design. AFR: African, ARIC: Atherosclerosis Risk in Community Study, EUR: European, FDR: false discovery rate, HCHS/SOL: Hispanic Community Health Study/Study of Latinos, GWAS: genome-wide association study, HIS: Hispanic, LD: linkage disequilibrium, MR: Mendelian randomization, OR: odds ratio, PRACTICAL: Prostate Cancer Association Group to Investigate Cancer Associated Alterations in the Genome, SNP: single nucleotide polymorphism. Information on scoring criteria used to evaluate the strength of evidence is shown in **Figure S1**.

An overview of the study design for this investigation is provided in Fig. 1. Metabolomic GWAS summary statistics for 1,740 AFR and 1,500 EUR genetic ancestry individuals were obtained from the Atherosclerosis Risk in Community (ARIC) study, while summary statistics for 3,848 HIS individuals were obtained from the Hispanic Community Health Study/Study of Latinos (HCHS/SOL).(39, 40)

Initially established in 1985 to identify risk factors for subclinical atherosclerosis, ARIC is a community surveillance and prospective US-based cohort study that recruited 15,792 middle-aged African American and White adults in four locations (Forsyth County, NC; Jackon, MS; suburban Minneapolis, MN; and Washington County, MD).(41) HCHS/SOL is community-based US cohort which recruited 16,415 Hispanic/Latino adults from four cities (Bronx, NY; Chicago, IL; Miami, FL; and San Deigo CA) between 2008–2011 with the goal of understanding chronic disease incidence, prevalence, and risk factors.(42) In ARIC, AFR and EUR populations were initially defined based on self-report. Individuals who were +/−4 standard deviations outside ancestry-specific genetic clusters defined with principal components were subsequently excluded. Given that Hispanic/Latino represents an ethnicity with ancestral diversity, in HCHS/SOL, HIS participants were those self-reporting as Cuban, Puerto Rican, Dominican, Mexican, Central American, or South American background.

### Metabolite data and GWAS

Metabolites in both cohorts were quantified via untargeted metabolomic profiling on fasting serum samples collected at baseline through Ultrahigh Performance Liquid Chromatography-Tandem Mass Spectroscopy at Metabolon, Inc. (Durham, NC).(40, 43, 44) In ARIC, metabolomic profiling was conducted in two batches in 2010 and 2014, while in SOL, metabolomic profiling was conducted more recently in 2017, leading to a larger number of quantified metabolites. In each study population, metabolites missing in > 80% of samples were excluded. Remaining missing metabolite values were assumed to be below the limit of detection (LOD) and imputed to the lowest LOD observed for each metabolite. Following previously described metabolite quality control,(39, 40) metabolite GWAS were conducted for 250 metabolites in ARIC and 711 metabolites in HCHS/SOL, including amino acids (68/176 in ARIC and HCHS/SOL), carbohydrates (9/23), cofactors and vitamins (6/25), energy-related metabolites (5/10), lipids (113/316), nucleotides (11/35), peptides (17/32), and xenobiotics (21/94).

Each metabolite was standardized with an inverse normalization transformation in each population separately. In linear regression models, each metabolite was then regressed on for sex, age at sample draw, batch, and center, with HCHS/SOL additionally adjusting for the six aforementioned populations. In sensitivity analyses, we additionally adjusted metabolite GWAS for body mass index (BMI) and smoking status (however, these additional adjustments could not be applied to the previously generated PCa GWAS summary statistics). All residuals were inverse normalized and rank-based normalized with the Blom transformation and used for subsequent analyses. Genotype data were generated on the Affymetrix Array 6.0 for ARIC and the Illumina Multi-Ethnic Genotyping Array (MEGA) for HCHS/SOL and imputed to the TOPMed-r2 reference panel.(45) GWAS were performed using linear regression models, with each metabolite as the outcome and each genetic variant as the predictor, adjusting for the first ten principal components to account for population stratification. In ARIC, analyses were performed separately in AFR and EUR ancestry populations. In both ARIC and HCHS/SOL, analyses were performed in SUGEN to account for relatedness.(46) After excluding rare variants (minor allele frequency (MAF) ≤ 0.01), two metabolites in the EUR ancestry data had a genomic inflation factor (λ) ≥ 1.10 and were removed from subsequent analyses.

### PCa GWAS

Population-specific PCa GWAS summary statistics were obtained from the most recent GWAS meta-analysis from the Prostate Cancer Association Group to Investigate Cancer Associated Alterations in the Genome (PRACTICAL) Consortium, which included 19,391/61,608 AFR ancestry, 122,188/604,640 EUR ancestry, and 3,931/26,405 HIS male cases/controls over 21 years of age.(47) There was no overlap between metabolite and PCa GWAS study participants. GWAS were performed using logistic regression models adjusted for age, sub-study (when applicable), and up to 10 principal components to account for population stratification. Genomic coordinates were lifted over to build GRch38/hg38 with the liftOver R package to match exposure coordinates.(48)

### Metabolite genetic instruments

We developed genetic instruments separately in each population for each metabolite and limited to independent (r^2^ < 0.2) autosomal single-nucleotide polymorphisms (SNPs; indels were not included) associated with each metabolite at the genome-wide significance level (P<5×10^− 8^), excluding SNPs that were poorly imputed (info score < 0.60), rare (MAF ≤ 0.01), or not present in the PCa GWAS summary statistics. MAF were used to infer the leading strand during exposure and outcome data harmonization when possible. Ambiguous palindromic SNPs were removed and replaced with the non-palindromic SNP in the linkage disequilibrium (LD) block with the next smallest p-value. For computational efficiency, LD pruning was performed separately in each population using LD estimates from TOP-LD, which included a subset of 1,335 AFR ancestry and 13,160 EUR ancestry individuals from TOPMed.(45, 49) As TOP-LD lacks HIS populations, LD pruning for HIS instruments was performed using LD estimated in EUR and then further pruned based on LD in AFR ancestry populations. In sensitivity analyses, statistically significant findings were further investigated with a more stringent LD threshold of r^2^ < 0.01 (rather than r^2^ < 0.2). Since TOP-LD only provides LD estimates for pairs of variants with r^2^ ≥ 0.2, sensitivity analyses of significant findings used LD calculated in TOPMed freeze 12 with 10,495 AFR ancestry, 10,864 EUR ancestry, and 8,100 HIS individuals. R^2^ estimates and F statistics were calculated for each SNP. All variants included in the metabolite instruments had an F statistic > 10. Statistical analyses were performed in R (version 4.4.0).

### Statistical analyses

#### MR analyses

In each population, MR analyses were performed via the TwoSample R package,(50) with inverse variance weighted (IVW) random effect results presented as primary analyses; Wald ratios were presented when an instrument included only one SNP. IVW fixed, IVW random multiplicative, weighted median, weighted mode, MR Egger, and MR PRESSO(51) (when instruments contained ≥ 10 SNPs) results were presented as sensitivity analyses to evaluate violations of MR assumptions, including: i) instruments are robustly associated with the outcome (relevance assumption), ii) instruments are not associated with confounders (independence assumption), and iii) instruments only associate with the outcome via the exposure (exclusion restriction assumption). In brief, these MR methods combine the effect sizes of included SNPs (obtained from GWAS summary data) into a single instrumental variable, with IVW approaches weighting each SNP by the inverse of the variance of their effect and then meta-analyzing effects across all SNPs while sensitivity analyses use approaches with different relaxations of MR assumptions.(52–54)

Analyses were performed separately in each population to obtain population-specific MR results. For metabolites with a genetic instrument in ≥ 2 populations, we reported cross-population MR results, using fixed effect models to meta-analyze population-specific IVW random effects and evaluating heterogeneity with Cochran’s Q statistic in the meta R package(55). Random effects meta-analyses were additionally conducted for metabolites with i) evidence of heterogeneity (Q-test P_het_<0.05) and ii) MR instruments in all three populations. Statistical significance for exploratory metabolome-wide analyses was defined as false discovery rate (FDR) < 0.10 to account for multiple testing. MR results are reported following the Strengthening the Reporting of Observational Studies in Epidemiology using Mendelian Randomization (MR-STROBE) guidelines (**Additional File 1**).(56)

#### Pathway enrichment analyses

Pathway enrichment analyses were performed on significant MR results in each population and cross-population meta-analyses, with the corresponding set of tested metabolites used as the reference panel. Enrichment analyses were performed for super- and sub-pathways (defined by Metabolon; **Table S1**) with a one-sided Fisher’s exact hypergeometric test using the bc3net R package.(57) Statistical significance was defined as FDR < 0.05 to account for multiple testing.

### Evaluating strength of evidence

We evaluated the strength of MR evidence for metabolites significantly associated with PCa risk by expanding previously established criteria to select metabolites with strong evidence of associations with PCa risk and further investigate in follow-up analyses.(36) Each metabolite could receive up to nine points based on nine criteria focused on population-specific evidence, cross-population evidence, and replication evidence (**Figure S1**). For population-specific evidence, up to four points could be given for 1) FDR < 0.10, 2) no evidence of MR assumption violations, 3) nominal unadjusted P < 0.05 using the LD threshold of r^2^ < 0.01 for metabolite instruments, and 4) no evidence of MR assumption violations with stringent instruments. For cross-population evidence, up to two points could be given for 1) FDR < 0.10 in meta-analyses and 2) unadjusted P < 0.05 for stringent instruments. For replication evidence, up to three points could be given for 1) unadjusted P < 0.05 in a second population using the stringent instrument, 2) no evidence of MR assumption violations in the second population, and 3) a consistent effect direction in the second population. Metabolites with a score ≥ 4 were deemed as having strong MR evidence and followed up in additional analyses described below.

### Follow-up of metabolites with strong evidence

#### Colocalization

Colocalization was used to investigate the presence of shared causal variants between a metabolite and PCa risk, using population-specific GWAS summary statistics to test five hypotheses: H0: neither trait has a causal variant in the region; H1: only the metabolite has a causal variant in the region; H2: only PCa risk has a causal variant in the region; H3: both traits have a causal variant in the region but the causal variants are independent; and H4: both traits colocalize to the same causal variant in the region.(58) Regions were defined as +/−100kb windows on either side of each evaluated SNP. Colocalization analyses were conducted via the coloc R package with three default variant level prior probabilities: P1: 1e − 4 (probability each variant is causally associated with the metabolite only); P2: 1e − 4 (probability each variant is causally associated with PCa risk only); and P12: 1e − 5 (probability each variant is causally associated with both the metabolite and PCa).(59) Evidence of colocalization was defined as posterior probability of H4 (PP.H4) > 70% or PP.H4/(PP.H3 + PP.H4) > 70%. The latter represents the probability of colocalization given the presence of a causal variant for PCa and can be beneficial when low statistical power leads to low PP.H4 and PP.H3.(60–63)

#### Secondary evaluations of pleiotropy

We investigated whether our findings for metabolites with strong MR evidence could be impacted by pleiotropy, given the strong correlation structure of metabolomic data, by: 1) performing IVW multivariate MR (MVMR), which accounts for shared genetic variants across metabolites that could reflect correlation,(64–67) based on metabolite instruments using the r^2^ < 0.01 LD threshold, 2) evaluating whether genetic variants in metabolite instruments were most predictive of the respective vs another metabolite, similar to a previous investigation,(68) and 3) identifying traits previously associated with variants in our metabolite instruments. MVMR was conducted utilizing the MVMR R package.(67) The latter was accomplished by searching the GWAS catalog using LDtrait(69) from the LDlinkR R package for variants included in metabolite instruments or correlated with instruments (r^2^ ≥ 0.95 within a 500kb window based on matched populations from the 1000 Genomes Project Phase 3).(70) We also evaluated correlations between metabolite instrument variants and 439 autosomal variants from a previously developed multi-ancestry PCa polygenic risk score (PRS)(71) using LDmatrix from the LDlinkR R package. Metabolite instrument variants were annotated to the nearest gene and for functional consequence using wANNOVAR.(72)

#### Associations between identified metabolites and other cancers and traits

We investigated whether circulating levels of the identified metabolites were previously associated with cancers or other traits by searching the Exposome Explorer using metabolite names and all reported synonyms from HMDB. Searches were conducted on April 30, 2025.

#### Drug and diet metabolite targets

We investigated whether the identified metabolites could be drug modifiable by searching the DrugBank pharmaco-metabolomics database(73) using metabolite names and synonyms from HMDB. We investigated whether the identified metabolites have been previously quantified in foods and potentially modifiable by diet by searching FooDB using HMDB IDs and searching MetaboFood using metabolite chemical formulas and names. Searches were conducted on April 30, 2025.

## Results

### Population-specific MR

**Figure 2:**
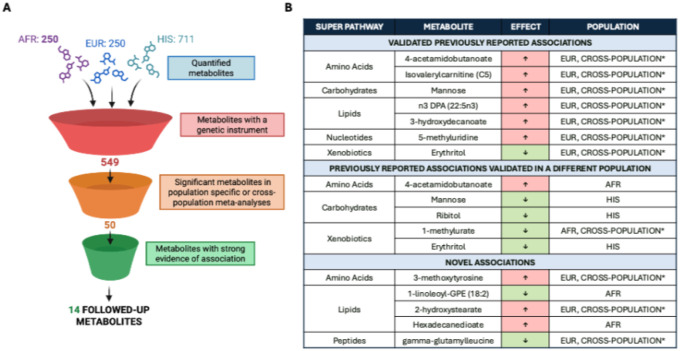
Overview of MR findings. **A:** Schematic highlighting the number of metabolites retained at each analysis stage, leading to the retention of 14 metabolites for follow-up analyses. **B**: Summary of findings for the 14 followed-up metabolites. ↑ indicates risk increasing association. ↓ indicates risk decreasing association. The previous PCa metabolomics MR studies referenced here were conducted in European populations (see **Table S5**). * No previous MR studies have reported Cross-population associations.

We evaluated 732 associations between 549 serum metabolites (146 amino acids, 20 carbohydrates, 13 cofactors and vitamins, 9 energy metabolites, 258 lipids, 26 nucleotides, 31 peptides, and 46 xenobiotics) and PCa risk, including 131, 106, and 495 metabolites tested in AFR, EUR, and HIS, respectively (Fig. 2 and **Figure S2**).

**Figure 3:**
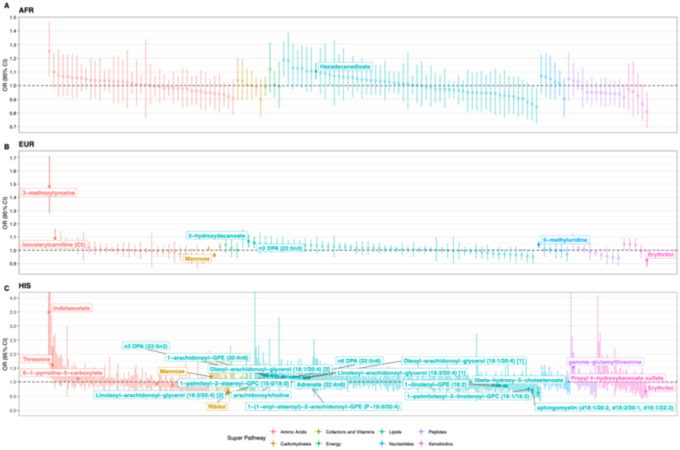
Population-specific PCa metabolomics MR results. Results are shown for IVW random effect MR in **A:** African ancestry, **B:** European ancestry, and **C:** Hispanic populations. Significantly associated (FDR<0.10) metabolites with no evidence of MR assumption violations in sensitivity analyses are named and have shaded circles.

In total, 45 unique metabolites were significantly associated with PCa risk across population-specific MR analyses (Fig. 3 and **Tables S1-S3**). In AFR, one of the 131 tested metabolites, hexadecanedioate (a fatty acid dicarboxylate), was significantly associated with PCa risk (OR = 1.10, 95% CI = 1.05–1.16, P_adj_=0.02), with no evidence of pathway enrichment observed (**Table S4**). In EUR, seven of the 106 tested metabolites (two amino acids, one carbohydrate, two lipids, one nucleotide, and one xenobiotic) were significantly associated with PCa risk, with the strongest association observed for the amino acid 3-methoxytyrosine (OR = 1.48, 95% CI = 1.28–1.71, P_adj_=8.7×10^− 6^) and no evidence of any pathway enrichment observed. In HIS, 40 of the 495 tested metabolites (five amino acids, two carbohydrates, one cofactor and vitamin, 29 lipids, one peptide, and two xenobiotics) were significantly associated with PCa risk. These metabolites were enriched for the lipid super pathway (P_adj_=0.007) and the polyunsaturated fatty acids (PUFAs) (n3 and n6) (P_adj_=0.001) and phosphatidylcholine (P_adj_=0.005) sub-pathways.

While no evidence of MR assumption violations was identified in AFR and EUR populations, 17 of the identified metabolite associations in HIS displayed evidence of MR assumption violations (**Table S1, Figure S1**). Among metabolites without evidence of MR assumption violations in HIS, the strongest association was observed for the xenobiotic propyl 4-hydroxybenzoate sulphate (OR = 1.23, 95% CI = 1.17–1.34, P_adj_=3.75×10^− 9^) (Fig. 3 and **Table S1**).

### Cross-population MR

**Figure 4:**
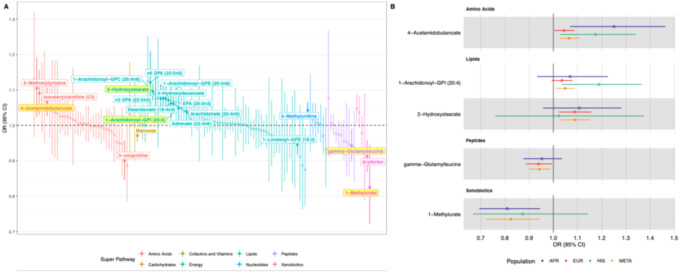
Cross-population PCa metabolomics MR results. **A:**Meta-analysis of population-specific IVW random effects MR estimates. Significantly associated (FDR<0.10) metabolites are named and have shaded circles. Name labels with yellow shading were significant in the cross-population MR but not population-specific MR. **B:** Comparison of effects for metabolites that were significant in cross-population but not population-specific MR. Shaded circles represent significance (FDR<0.10).

Of the metabolites investigated, 131 had genetic instruments in > 1 population, of which 52 had genetic instruments in all three populations (**Tables S1-S3**). In cross-population meta-analyses of these metabolites, 21 (four amino acids, one carbohydrate, 12 lipids, one nucleotide, one peptide, and two xenobiotics) were significantly associated with PCa risk, 11 of which had genetic instruments in all three populations (Fig. 4 and **Table S1**). The strongest association observed in cross-population analyses was for 1-arachidonoyl-GPC (20:4n6) (OR = 1.10, 95% CI = 1.08–1.12, P_adj_=2.4×10^− 19^) (Fig. 4 and **Table S1**). The 21 metabolites were significantly enriched for the PUFA (n3 and n6) sub-pathway (P_adj_=0.003; **Table S4**). Five of the 21 metabolites were newly identified in cross-population MR meta-analyses (1-methylurate, 1-arachidonoyl-GPI (20:4), 2-hydroxystearate, 4-acetamidobutanoate, and gamma-glutamylleucine), while three metabolites identified in population-specific MR analyses were not significant in cross-population MR meta-analyses (hexadecanedioate, indoleacetate, and threonine).

Across all metabolites meta-analyzed, we found evidence of heterogeneity (Q-test P-value < 0.05) across populations for 22 metabolites in fixed effect meta-analyses (**Table S1**), including 12 of the 21 metabolites significantly associated with PCa risk in cross-population MR. Of the 22 metabolites with evidence of heterogeneity, 11 had genetic instruments in all three populations and could therefore be meta-analyzed using random effects meta-analyses, including 8 of the 21 metabolites significantly associated in cross-population analyses. Of these eight initially significant metabolites, two remained significant using random effects models (4-acetamidobutanoate and 1-linoleoyl-GPE (18:2); **Table S5**).

**Figure 5:**
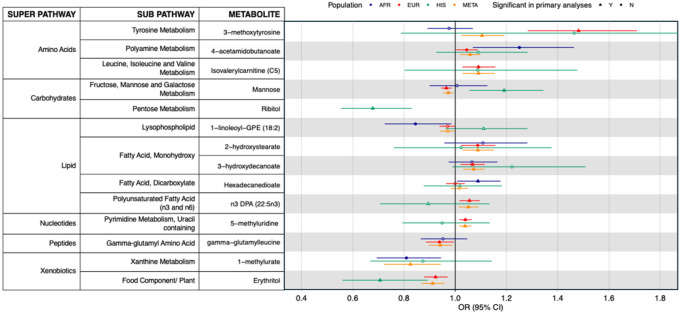
MR results for metabolites with strong evidence of association with PCa risk. Results are shown for IVW random effects MR estimated based on the stringent LD threshold of r^2^ <0.01 used in sensitivity analyses. Shaded shapes indicate an unadjusted P<0.05. Triangles indicate associations that were significant in primary analyses using an LD threshold of r^2^<0.2.

In total, 50 unique metabolites were significantly associated (FDR < 0.1) with PCa risk in population-specific or cross-population analyses. For these metabolites, results from sensitivity analyses using metabolite GWAS summary statistics additionally adjusted for BMI and current smoking status were highly correlated with our primary results (beta r = 0.97, P-value r = 0.96 **Figure S5**). In additional sensitivity analyses, MR instruments were developed for these 50 metabolites using a more stringent LD threshold of r^2^ < 0.01 in all populations with an available metabolite instrument. Of the initially identified significant associations, 1/1, 7/7,7/40, and 17/21 associations remained significant in AFR, EUR, HIS, and cross-population analyses, respectively, using the stringent LD threshold (Fig. 5, **Table S6**).

### Strength of evidence

In evaluating the strength of evidence across association, replication, and sensitivity analyses (criteria described in the Methods), 14 metabolites had strong MR evidence including three amino acids (3-methoxytyrosine, 4-acetamidobutanoate, and isovalerylcarnitine (C5)), two carbohydrates (mannose and ribitol), five lipids ((1-linoleoyl-GPE, 2-hydroxystearate, 3-hydroxydecandoate, hexadecanedioate and n3-DPA (22:5n3), one nucleotide (5-methyluridine), one peptide (gamma-glutamylleucine) and two xenobiotics (1-methylurate and erythritol) (**Figure S1**). With regards to previous metabolite-PCa MR findings, five of these were novel (1-linoleoyl-GPE, 2-hydroxystearate, 3-methoxytyrosine, gamma-glutamylleucine, and hexadecanedioate) and nine validated previous MR findings (Fig. 2, **Table S7**).

### Follow-up analyses

#### Colocalization

Among the 14 metabolites with strong MR evidence, 22 genetic regions were investigated for colocalization with PCa risk. Based on the PP.H4 > 70% criteria, evidence of colocalization was observed for one of the three SNPs (rs28864441) in the AFR hexadecanedioate instrument (PP.H4 = 0.759) (**Figure S4** and **Table S8**). Based on the PP.H4/(PP.H3 + PP.H4) > 70% criteria, loci in six additional metabolites displayed evidence of colocalization: 2-hydroxystearate, 5-methyluridine, and isovalerylcarnitine (C5) based on EUR instruments, erythritol and ribitol based on HIS instruments, and mannose based on both EUR and HIS instruments.

#### Secondary evaluations of pleiotropy

Several secondary analyses were conducted to further investigate the extent and potential impact of pleiotropy for the 14 metabolites with strong MR evidence. MVMR analyses were performed in each population where the genetic instrument for any of the 14 metabolites (utilizing the r^2^ < 0.01 LD threshold) demonstrated strong evidence. In MVMR analyses, of the metabolites initially associated in population-specific MR analyses, hexadecanedioate in AFR, 3-methoxytyrosine and mannose in EUR, and mannose and ribitol in HIS were associated with PCa risk (**Figure S5**).

The metabolites that were not significant in MVMR analyses likely reflect shared pathways with the significantly associated metabolites, particularly among metabolites associated with PCa risk in EUR ancestry individuals.

Each of the 22 variants (**Table S10)** included in the 14 metabolite instruments were typically the strongest predictor of the respective metabolite whose instrument they belonged to (**Figures S6-S8**). The few exceptions were rs77271279 in the AFR hexadecanedioate instrument, which was a stronger predictor of glycocholenate sulfate; rs174599 in the EUR n3-DPA (22:5n3) instrument, which was a stronger predictor of 10 other mostly phospholipid metabolites, as expected since this variant is in the highly pleiotropic fatty acid desaturase (*FADS*) region; and rs72692616 in the HIS erythritol instrument, which was a stronger predictor of ribitol, which was associated with decreased PCa risk in HIS, similar to erythritol. However, these three instruments each included 1–2 additional variants, each of which were the strongest predictor of the given metabolite.

In LDtrait, 18 of the 22 variants included in the 14 metabolite instruments were previously associated with an average of 87, 168, and 346 traits in AFR, EUR, and HIS populations, respectively (**Figure S9, Table S11**), No associations were identified in LDtrait for the EUR gamma-glutamylleucine instrument or the AFR 4-acetamidobutanoate instrument. Most associations identified in LDtrait were with metabolite phenotypes, with 12 of the 22 variants previously associated with non-metabolite phenotypes, including alcohol use and dependency (2 variants), adiposity-related traits (3 variants), male hormone-related traits (3 variants), and cancer outcomes (3 variants) (**Table S12**). Several variants demonstrated a high degree of pleiotropy, including missense variant rs1260326 in the EUR and HIS mannose instrument (associated with 1001 unique traits aside from mannose in both populations, including alcohol-, adiposity-, and hormone-related traits), rs35246381 in the EUR 4-acetamidobutanoate instrument (244 traits, including bladder cancer), rs174570 in the AFR 1-linoleoyl-GPE (18:2) instrument (215 traits, including skin cancer and sex hormone-binding globulin levels), and rs174599 in the EUR n3-DPA (22:5n3) instrument (190 traits, including colorectal cancer and male pattern baldness). The latter two variants are in the *FADS* region.

Metabolite instrument variants had low correlations with PCa PRS variants, with most pairwise correlations having an r^2^ < 0.18, except for rs11043003 in the EUR 3-methoxytyrosine metabolite instrument and rs7123299 in the PCa PRS (r^2^ = 0.41; **Table S13**).

#### Metabolites associated with cancer outcomes

Using Exposome Explorer, we found that two of the 14 metabolites were previously associated with cancer outcomes: 1-methylurate was associated with increased brain cancer risk, while n3-DPA (22:5n3) was associated with decreased colorectal cancer risk and increased prostate cancer risk (**Table S14**), consistent with our observed positive association between n3-DPA (22:5n3) and PCa risk in EUR and cross-population analyses.

#### Drug and diet targets

**Figure 6:**
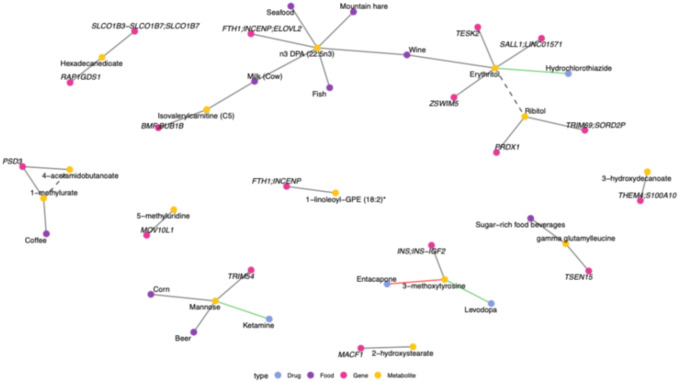
Metabolites associated with PCa risk and their corresponding genes, dietary factors, and drugs. Metabolites with strong evidence of association with PCa risk are shown in the network plot. Gene names (pink) represent the nearest genes for variants associated with the metabolite. Dietary exposures (purple) for each metabolite were identified in FooDB, MetaboFood, and Exposome explorer. When numerous types of the same food category were identified (e.g., species of fish), foods were grouped for clarity. Drug exposures (blue) were identified in the DrugBank pharmacometabolomics database. Red lines from drug nodes indicate drug associations that decrease metabolite levels. Green lines from drug nodes indicate drug associations that increase metabolite levels. Dashed lines between metabolites indicate pleiotropy between metabolite genetic instruments identified in LDtrait.

Using DrugBank, we found that three of the 14 metabolites were modifiable by drugs (Fig. 6, **Table S14**). Levels of 3-methoxytyrosine decrease and increase in response to levodopa (L-DOPA) and entacapone, respectively, both of which are used to treat Parkinson’s disease. Erythritol levels increase with use of the blood pressure medication hydrochlorothiazide. Mannose levels increase with the administration of the general anesthetic ketamine.

We also investigated whether the 14 metabolites could be modified by diet. Using FooDB, four metabolites were previously quantified in food, with erythritol and mannose quantified in wine and beer, respectively, isovalerylcarnitine (C5) quantified in cow milk, and n3-DPA (22:5n3) quantified in 119 protein and dairy products. Nine others were “expected” to be but not yet quantified in foods (Fig. 6, **Table S14**). Two additional associations were identified using MetaboFood and Exposome Explorer, with 1-methylurate previously associated with coffee and gamma-glutamylleucine associated with sugar-rich food beverages (Fig. 6, **Table S14**). Likewise, using LDtrait, we found that variants in the mannose, erythritol, ribitol, and hexadecanedioate instruments were previously associated with alcohol consumption and variants in the mannose instruments were previously associated with coffee consumption (**Tables S10-S11**).

## Discussion

We conducted a comprehensive and exploratory large-scale cross-population untargeted metabolomics-PCa MR, leading to the identification of 50 metabolites demonstrating significant associations with PCa risk, 14 of which had strong evidence after considering sensitivity analyses and evidence across populations. Of these, five were potentially novel biomarkers of overall PCa risk and seven validated previous findings. In extensive follow-up analyses evaluating potential environmental modifiers of these 14 metabolites, at least three were targets for existing drugs and six have been quantified in or associated with dietary factors, suggesting that these PCa risk metabolites may be modifiable. These findings provide insights into potentially causal mechanisms leading to the development of PCa and could have implications for prevention and screening strategies.

Five of the 14 metabolites with strong evidence of association with PCa risk were lipids, including a positive association with the long-chain omega-3 PUFA n3-DPA (22:5n3). Further, HIS and cross-population results demonstrated enrichment for omega-3 and omega-6 PUFAs. PUFAs have long been associated with numerous health outcomes, including cancer, and fatty acid dysregulation is known to help facilitate the migration and proliferation of cancer cells.(25, 74) While omega-3 PUFAs typically show health-promoting effects, consistent with our findings, a European ancestry MR reported that n3-DPA (22:5n3) was associated with increased PCa risk,(25) while observational studies and meta-analyses have shown modest positive associations between omega-3 PUFAs and PCa risk.(75–78) However, another study reported that omega-3 PUFAs (measured post-diagnostic in cases) were inversely associated with PCa in Ghanaian men.(79) Although PUFAs were not available in our AFR data to evaluate these associations, these findings could reflect historical dietary selection pressures that led to population differences in PUFA metabolism,(80, 81) different dietary sources of omega-3 PUFAs, or different screening practices. It has been suggested that positive associations between omega-3 PUFAs and PCa risk could reflect healthier individuals being more likely to undergo screening and receive a PCa diagnosis.(82, 83) While our causal inference approach makes it less likely for findings to be impacted by such screening selection bias, it will be important for future studies to evaluate PUFAs across global populations where screening practices vary and with consideration of lethal PCa risk. Further, many of the PUFAs we identified displayed evidence of pleiotropy, which was expected given the versatile role of PUFAs in human health and their link to the highly pleiotropic *FADS* locus.(15, 84) Our findings may reflect vertical pleiotropy, where instruments are associated with multiple traits in the same biological pathway (not violating MR assumptions); however, given the potential for horizontal pleiotropy, where instruments are associated with traits in different pathways (violating MR assumptions), our PUFA findings should be interpreted with caution.

In addition to n3-DPA (22:5n3), we identified three additional fatty acids (hexadecanedioate, 3-hydroxydecanoate and 2-hydroxystearate) associated with increased PCa risk. Hexadecanedioate, a fatty acid dicarboxylate, is a novel association identified in AFR ancestry individuals. This finding was strengthened by evidence of significant colocalization for rs28864441, located in the *RAP1GDS1* gene, expression of which has been shown to be upregulated in PCa tissue.(85) Hower, hexadecanedioate was not significantly associated with PCa risk in EUR or HIS in our study, despite post-hoc power calculations(86) demonstrating hexadecanedioate instruments having an 80% power to detect effect sizes of similar magnitude (EUR OR ≥ 1.03, HIS OR ≥ 1.12) as observed in AFR (OR = 1.10). Previous MR studies in European descent populations have also reported null associations between hexadecanedioate and PCa risk;(27, 28, 32, 33) however, an observational investigation in a predominantly non-Hispanic White population found that hexadecanedioate was associated with increased risk of overall and aggressive PCa.(87) Hexadecanedioate, a product of fatty acid β-oxidation, is a suspected marker of impaired mitochondrial β-oxidation and dysregulated lipid metabolism.(88) Dicarboxylate fatty acids (quantified as tetradecanedioate and hexadecanedioate) have also been shown to be significantly increased in tumors resistant to androgen deprivation therapy compared to non-resistant tumors.(89) Interestingly, population differences in plasma hexadecanedioate levels were observed in a small (N = 60) breast cancer metabolomics study that reported significantly higher levels in African American compared to White American individuals and in cases compared to controls.(90) Overexpression of the mitochondrial and peroxisomal enzyme alpha-methylacyl-CoA racemase (AMACR), involved in fatty acid β-oxidation, is also well established to be associated with increased PCa risk(91, 92). Future work is needed to delineate how metabolites involved in fatty acid β-oxidation contribute to PCa pathogenesis and risk across populations.

This investigation also highlighted associations between two monohydroxylated fatty acids: 3-hydroxydecanoate, previously associated with increased PCa risk in a European ancestry MR study(33), and a novel positive association between 2-hydroxystearate and PCa risk not identified in previous observational(19) or MR studies. Supporting this finding, we identified evidence of colocalization for rs7529794 in the 2-hydroxystearate instrument, located in the *MACF1* gene—a cytoskeleton regulator that regulates key cellular processes, including cellular migration and proliferation. Although no studies to date have investigated the role of *MACF1* in PCa risk, *MACF1* upregulation has been reported in breast, lung, and brain cancers.(93) Hydroxylation of fatty acids can considerably alter the physiological properties of fatty acids, positioning them as key components in membrane structure and cellular signaling.(94, 95) Although investigations into the role of hydroxylated fatty acids in cancer are limited, increased expression of glycosphingolipids containing high levels of hydroxylated fatty acids have been identified in a range of tumor types.(94)

Our investigation also identified a novel association between the lysophospholipid 1-linoleoyl-GPE (18:2) and reduced PCa risk. Corroborating this finding, our recent systematic review of untargeted prospective metabolomic studies identified an inverse association between 1-linoleoyl-GPC (18:2) (a structurally similar lysophospholipid with a choline head group in place of the ethanolamine head group) and lethal PCa risk.(19) Lysophospholipids are lipid synthesis intermediates, signaling mediators and immune response modulators that have been associated with cancer development and the tumor microenvironment,(96) and differences in 1-linoleoyl-GPE (18:2) concentrations between the peripheral (where ~ 70% of PCa is diagnosed) and transitional anatomical glandular zones in the prostate have been reported in non-cancerous tissue.(97) Furthermore, lysophospholipids can be converted to lysophosphatidic acid (LPA),(98) which in turn can bind to LPA receptors, the expression of which has been shown to impact PCa development and progression.(99) Future work is warranted to better understand the role of specific monohydroxylated fatty acids and lysophospholipids in PCa development.

Alongside lipids, our investigation identified nucleotide metabolites associated with PCa risk, including a positive association between the pyrimidine nucleoside 5-methyluridine and PCa risk in the EUR population, in agreement with previous untargeted European MR studies.(27, 32, 33) Pyrimidine nucleosides are essential in DNA replication and increased extracellular pyrimidine uptake increases DNA replication in malignant cancers.(100) Our recent systematic review found positive associations between three pyrimidine nucleotides, 2-O-methyluridine, 5,6-dihydrouridine, and dihydroorotate, and lethal PCa risk.(19) Although observational findings for 5-methyluridine have been mixed,(101–103) *in vitro* analyses found higher extracellular 5-methyluridine levels in the environment of PCa cells compared to non-tumoral cells(104) and increased gene expression dysregulation in the pyrimidine metabolism pathway in lethal compared to non-lethal cases.(105) Of note, our observed association between 5-methyluridine and PCa risk was attenuated in MVMR analyses and was not observed in Hispanic individuals. Additional investigations into the role of 5-methyluridine as an independent PCa biomarker in diverse populations are warranted.

Our analyses also identified an inverse association between the xenobiotic erythritol (often utilized as an artificial sweetener)(106, 107) and PCa risk, corroborating previous evidence reported for prostate(32, 33) and breast cancer.(27) Erythritol is a sugar alcohol involved in nucleic acid and nicotinamide adenine dinucleotide phosphate (NADPH) synthesis that is produced from glucose via the pentose phosphate pathway (PPP), a recognized target pathway in cancer prevention.(107, 108) While previous observational circulating metabolomic investigations have not identified an association between erythritol and PCa risk, a urinary study found significantly lower levels of erythritol in PCa patients compared to controls.(109) We also identified an inverse association for the carbohydrate ribitol, involved in pentose metabolism, and PCa risk in HIS individuals. Like erythritol, ribitol is a sugar alcohol and PPP intermediate. It has previously been shown to enhance the ability of the cancer therapeutic JQ1 to inhibit cellular proliferation and migration of certain breast cancer cell lines.(110) Because ribitol only had a HIS genetic instrument, we could not examine cross-population effects; however, previous MR studies in European descent populations have identified an inverse effect of ribitol on PCa risk.(32, 33) Future work should assess the role of ribitol and PPP metabolites in PCa risk across additional diverse populations.

We observed population-specific effects for the carbohydrate mannose, which was inversely associated with PCa risk in EUR and positively associated with PCa risk in HIS. Previous MR studies have also found mannose to be associated with decreased PCa risk in European descent individuals,(31–33) and *in vitro* analyses have found mannose to have anti-proliferation and pro-apoptotic effects.(111) The opposing effect directions we observed in EUR and HIS populations are likely due to SNPs included in the mannose instrument having opposing effects in the EUR and HIS PCa GWAS (**Figure S10**). Future mechanistic and epidemiologic studies investigating the role of mannose in PCa development across populations is warranted to validate potential population-specific effects.

This study has several strengths. It is the largest PCa metabolomics MR investigation to date with regards to the number of metabolites investigated and the PCa sample sizes included. Moreover, it is the first PCa metabolomics MR study to include multiple genetic ancestry groups and the first to investigate African ancestry populations, which is notable given the high risk of PCa in this population. We conducted comprehensive sensitivity analyses to assess MR assumption violations and follow-up analyses to highlight potential environmental modifiers of identified metabolites, providing additional biological and clinical context to our findings. Nonetheless, this study also has limitations. Firstly, although all included cohorts utilized the Metabolon platform, metabolomics profiling was conducted at different timepoints with different platform versions in each population, reducing the comparability and availability of metabolites across populations and our ability to investigate cross-population effects. Moreover, although this is the only diverse PCa metabolomics MR investigation to date, findings may not be generalizable across all populations meaning additional studies are warranted in other populations. HCHS/SOL had the largest number of metabolites measured and sample size for the metabolite GWAS, resulting in more associated metabolites identified in the HIS population. Secondly, to maximize statistical power, metabolite GWAS were conducted across sexes; although metabolite GWAS were adjusted for sex, residual differences in metabolism between sexes could impact results.(22, 26, 27, 34) Thirdly, some metabolite instruments only contained one SNP, meaning it was not possible to fully assess pleiotropy. Finally, as this investigation was based on serum samples, we may not have captured plasma-specific or tissue-specific associations between metabolites and cancer risk.(27, 112)

This large-scale MR of pre-diagnostic serum metabolomics highlights the role of numerous metabolites, including lipids, amino acids, and nucleotides, in PCa risk across diverse population groups. Many of these metabolites are biologically relevant to PCa pathogenesis, potentially offering novel mechanistic insights, and some have been previously associated with cancer risk, including prostate, endometrial, ovarian, and breast cancer while others are potentially novel. In evaluating the clinical utility of these metabolites as PCa biomarkers across populations, future work should assess the role of these metabolites in high-risk and lethal PCa, particularly given previous observations of metabolite associations varying by PCa aggressiveness.(19) Functional validation via *in vitro* or *in vivo* experiments could further elucidate the mechanisms through which these metabolites may impact the development of PCa.

## Supplementary Files

This is a list of supplementary files associated with this preprint. Click to download.

- Appendix1STROBEMRchecklist.docx
- MRsupplementtablessubmitted.xlsx
- MRsupplementsubmitted.docx

## Data Availability

Datasets supporting the conclusions of this article included within the article and its supplementary material. The prostate cancer GWAS summary statistics are available in the GWAS Catalog (https://www.ebi.ac.uk/gwas/) under the following accession codes: European (GCST90274714), African (GCST90274715), and Hispanic (GCST90274717). The metabolite GWAS summary statistics generated for this investigation will be available in the GWAS Catalog upon publication (accession number xxx).

## Abbreviations

AFR: African
ARIC: Atherosclerosis Risk in Community study
BMI: body mass index
CI: confidence interval
EUR: European
GWAS: genome-wide association study
FADS: fatty acid desaturase
FDR: false discovery rate
HCHS/SOL: Hispanic Community Health Study/ Study of Latinos
HIS: Hispanic
IVW: inverse variance weighted
LD: linkage disequilibrium
LOD: limit of detection
MAF: minor allele frequency
MEGA: Multi-Ethnic Genotyping Array
MR: Mendelian randomization
MR-STROBE: Strengthening the Reporting of Observational Studies in Epidemiology using Mendelian Randomization
MVMR: multivariate MR
OR: odds ratio
PCa: prostate cancer
PPP: pentose phosphate pathway
PUFA: polyunsaturated fatty acid
PRACTICAL: Prostate Cancer Association Group to Investigate Cancer Associated Alterations in the Genome
SNP: single-nucleotide polymorphism
